# NO_2_ Selective Sensor Based on α-Fe_2_O_3_ Nanoparticles Synthesized via Hydrothermal Technique

**DOI:** 10.3390/s19010167

**Published:** 2019-01-05

**Authors:** Mokhtar Hjiri, Mohamed Salah Aida, Giovanni Neri

**Affiliations:** 1 Department of Physics, Faculty of Sciences, King Abdulaziz University, 21589 Jeddah, Saudi Arabia; meida@kau.edu.sa; 2 Center of Nanotechnology, King Abdulaziz University, 21589 Jeddah, Saudi Arabia; 3 Department of Engineering, University of Messina, I-98166 Messina, Italy; gneri@unime.it

**Keywords:** α-Fe_2_O_3_, hydrothermal, NO_2_, selectivity, p-type behavior

## Abstract

In the present work, hematite (α-Fe_2_O_3_) nanopowders were successfully prepared via a hydrothermal route. The morphology and microstructure of the synthesized nanopowders were analyzed by using scanning and transmission electron microscopy (SEM and TEM, respectively) analysis and X-ray diffraction. Gas sensing devices were fabricated by printing α-Fe_2_O_3_ nanopowders on alumina substrates provided with an interdigitated platinum electrode. To determine the sensor sensitivity toward NO_2_, one of the main environmental pollutants, tests with low concentrations of NO_2_ in air were carried out. The results of sensing tests performed at the operating temperature of 200 °C have shown that the α-Fe_2_O_3_ sensor exhibits p-type semiconductor behavior and high sensitivity. Further, the dynamics exhibited by the sensor are also very fast. Lastly, to determine the selectivity of the α-Fe_2_O_3_ sensor, it was tested toward different gases. The sensor displayed large selectivity to nitrogen dioxide, which can be attributed to larger affinity towards NO_2_ in comparison to other pollutant gases present in the environment, such as CO and CO_2_.

## 1. Introduction

Metal oxides, such as ZnO, NiO, SnO_2_, and others, have been used for a long time as an active layer in gas sensors to detect toxic and hazardous environmental gases, such as CO, CO_2_, and NO_2_, because they have a low cost in comparison to other sensing technologies, are robust, have a small size, and are lightweight. Recently, the growth of industries and the improvements in quality of life have led to the increased importance of the problem of air pollution. As a consequence, the quality of urban air has lately become a problem of public health. Among the air pollutants, nitrogen dioxide (NO_2_) is one of the most harmful and highly toxic gases [1,2]. The sources of nitrogen dioxide are car exhausts and house and industrial combustion processes. The threshold limit value of NO_2_ is up to 25 ppm [3]; hence, its detection at this low concentration is necessary.

Widely used commercial gas sensors have, however, severe limitations. These concerns originate from their large power consumption, low sensitivity, and high working temperature [4,5]. Therefore, there is an urgent demand for new gas sensors with better performances. For this purpose, several metallic oxides have attracted increasing interest and are intensively investigated, such as ZnO [6], SnO_2_ [7], CdO [8], NiO [9], and Fe_2_O_3_ [10] for gas sensing. However, more research is still necessary to develop further improvements in gas-sensing properties, especially the sensitivity and selectivity [11]. These characteristics are of utmost importance for environmental applications where the target gas is usually present in low concentrations in a complex gaseous mixture.

Conductometric gas sensors can be fabricated by printing a film of the sensing material on the sensor substrate coated with interdigitated electrodes [12]. It is well known that the sensor performance is closely related to the morphology of the sensor surface exposed to the target gas. Obviously, sensors based on nanopowders perform better than thin, film-based ones; this is mainly due to the larger specific surface and reacting sites available in the powder in comparison to thin films, where the surface roughness is a limiting parameter.

Among metal oxide semiconductors, α-Fe_2_O_3_ is a good candidate as a sensing layer for different gases. α-Fe_2_O_3_ is an n-type metal oxide semiconductor with a band gap of 2.1 eV [13]. Iron oxide has been a commonly studied transitional metal oxide material due to its variable oxidation state, low cost, and remarkable magnetic properties [14,15]. Several methods have been used to synthetize α-Fe_2_O_3_ nanomaterials with different morphologies and nanostructures, including solvothermal synthesis [16], sol-gel method [17], thermal decomposition method [18], hydrothermal synthesis [19], and sonoelectrochemical anodization method [20]. Among these techniques, hydrothermal route is considered advantageous because of its simplicity and low cost [21].

Many reports about the detection of different gases and volatile organic compounds, such as NO_2_ [22], ethanol [23], H_2_S [24], and acetone [25], by pristine or doped α-Fe_2_O_3_ can be found in literature. Nowadays, many efforts have been aimed at enhancing the gas-sensing performance of α-Fe_2_O_3_ sensors by controlling its microstructure through many physical/chemical preparation techniques. Recently, it has been outlined that the hematite morphology plays an important role on gas-sensing properties. α-Fe_2_O_3_ prepared as thin films showed a selective detection for NO_2_ [22], while α-Fe_2_O_3_ nanowires are more sensitive to CO gas [26], and flowerlike α-Fe_2_O_3_ exhibited better performance regarding ethanol [27]. This shape dependence of sensor sensitivity towards gases is attributed to the variation of the available nanocrystal facets in contact with target gas [28]

On this basis, in this work, we report efforts made in synthesizing α-Fe_2_O_3_ nanoparticles by hydrothermal method, focusing on the development of a selective sensor for NO_2_ gas for applications in the field of environmental monitoring.

## 2. Experimental Procedure

### 2.1. Preparation of α-Fe_2_O_3_ Nanoparticles

The hydrothermal route was used to prepare Fe_2_O_3_ nanoparticles. The starting material is FeCl_3_ powder. This precursor was dissolved in water after magnetic stirring for 1 h to obtain a 0.1 M solution. Then, 2 mL of ammonia was added to the solution. The final solution was poured into a Teflon lined steel autoclave which was heated in a programmed furnace at 200 °C for 10 h. The resultant material was washed several times with a mixture of ethanol and water for purification and then dried at 60 °C for 1 h in an oven. Then, the obtained powder was annealed for 2 h at 500 °C.

### 2.2. Characterization

The microstructure of the material was analyzed using the X-ray diffraction technique (XRD, D8 Advance, manufacturer Bruker Corporation, Brucker, Germany). Scanning and transmission electron microscopy (SEM (Zeiss, Oberkochen, Germany) and TEM (JEOL. LTD., Tokyo, Japan), respectively) were used to evaluate the size and morphology of the particles. To perform SEM analysis, nanoparticles were attached onto an SEM holder by a carbon tape, utilizing an acceleration voltage of 15 kV. TEM analysis was performed by using a JEOL JSM-200F atomic resolution microscope (JEOL Legacy, Peabody, MA, USA) operating at 200 kV. Small amount of nanopowdered samples were dispersed in ethanol by using ultrasonic bath (type Branson CPX5800H-E, GT Sonic, Meizhou city, China) for several minutes, and the solution was dropped onto a carbon-coated Cu grid and left to dry in a clean room at room temperature.

### 2.3. Preparation of Sensor

First, a proper amount of α-Fe_2_O_3_ nanoparticles was mixed with several drops of distilled water until a paste was formed. The sensor was prepared by coating paste on a substrate of alumina, which is chemically inert with respect to hematite and is considered a refractory material, allowing sensors to work at high operating temperatures. Geometric dimensions of the substrate were 6 mm × 3 mm. On the front side, platinum electrodes were fixed, and on the back side, platinum resistances were used as a thermal heater. Sensing tests were performed under a dry air flow. The sensor resistance data were collected in the four points mode using an Agilent 34970A multimeter (KEYSIGHT, Santa Rosa, CA, USA). The operating temperature range of the tested sample was from 150 to 300 °C. Tests were performed in an experimental test bench which operates at a controlled temperature and reads resistance measurements by varying the gas concentrations. The response of the sensor is calculated as R_0_/R for oxidizing gas, where R_0_ is the baseline resistance of the sensor in air, while R is the sensor resistance when the target gas is turned on. Dynamic characteristics, such as response time, τ_res_, defined as the time required for the sensor resistance to reach 90% of the equilibrium value after NO_2_ is injected, and recovery time, τ_rec_, taken as the time needed for the sensor resistance to reach 90% of the baseline value in air, were also evaluated.

## 3. Results and Discussion

### 3.1. Microstructure and Morphology

**Figure 1:**
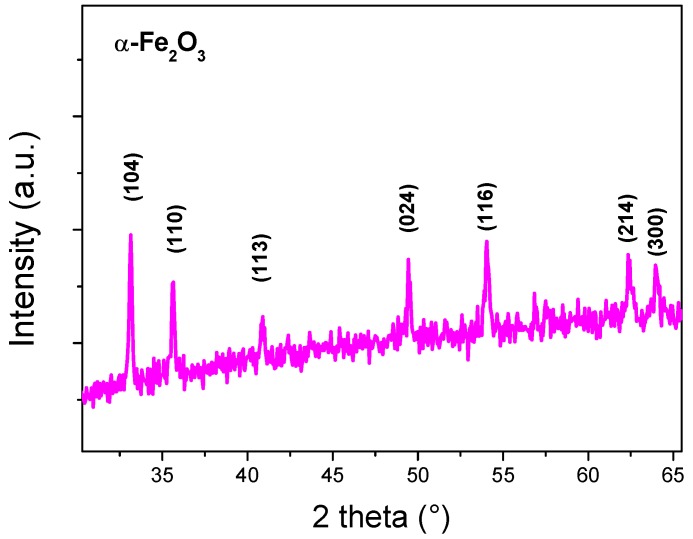
X-ray diffraction pattern of α-Fe_2_O_3_ nanoparticles.

Figure 1 shows the X-ray diffraction (XRD) pattern of the prepared sample annealed at 500 °C for 2 h. The diffraction peaks can be readily indexed to the rhombohedral (hexagonal) structures of hematite (JCPDS No: 33-0664) [29] with the lattice parameters a = b = 5.0387 Å and c = 13.767 Å. The peaks of hematite phase are located at 2θ = 24.11°, 33.16°, 35.56°, 40.87°, 49.43°, 53.96°, 62.46°, and 64.02°; they are assigned to (012), (104), (110), (113), (024), (116), (214), and (300) planes, respectively. No other phases were observed, indicating the purity of the prepared powder and the α-Fe_2_O_3_ single phase. No diffraction peak of the FeOOH phase was detected, indicating that the annealing had completely changed the FeOOH to α-Fe_2_O_3_ according to the following reaction:

2 FeOOH → α-Fe_2_O_3_ + H_2_O

The average crystallite size (*D*) of the particles is calculated by the Scherrer equation [30]:

$$D=\frac{0.9\lambda }{B\cos\theta_{B}}$$

where λ is the X-ray wavelength (1.5406 Å), *B* is the full width at half maxima (FWHM) of the concerned peaks, and *θ_B_* is the diffraction angle in degree. The most intense peak corresponding to (104) reflection was used to determine the crystallite size, which was estimated to be 55 nm. The average crystallite size is largely dependent on the synthesis method. Cuong et al. [28] have obtained a crystallite size of 40 nm in α-Fe_2_O_3_ nanopowder prepared by the same hydrothermal procedure of our work, followed by a heat treatment at 500 °C. However, they used ferric nitrate as the starting salt. As the crystallite size of the metal oxide products obtained is well known to depend on the type of salt used, this discrepancy might be due to the easier dissociation of chloride salt by comparison to nitrate ones, leading to different nucleation and growth kinetics. The same findings have been reported in the synthesis of other metal oxides. For example, Lehraki et al. have reported that ZnO prepared by using zinc chloride as a precursor displays large crystallite size, while a zinc nitrate precursor leads to smaller crystallite size [31].

**Figure 2:**
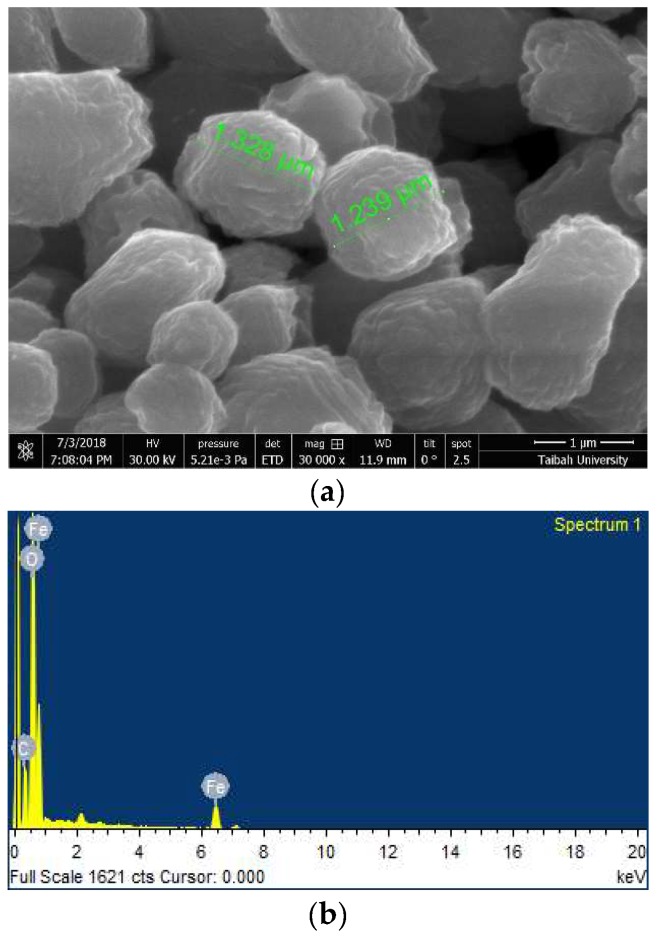
(**a**) Scanning electron microscopy (SEM) micrograph of α-Fe_2_O_3_ nanoparticles; (**b**) Energy dispersive x-ray EDX analysis.

The morphological properties of the sample have been investigated by SEM. The grains have irregular shapes, as shown in Figure 2a. The grains are formed by the coalescence of smaller grains. This may explain the observed irregular spherical and elongated shapes of the final grains. The same remarks have been outlined by several authors [32,33,34], highlighting that α-Fe_2_O_3_ powders prepared via the hydrothermal process are composed of particles of 1.5–2.5 μm in diameter because of the agglomeration of smaller nanoparticles.

Energy dispersive x-ray EDX analysis reported in Figure 2b indicates the presence of iron and oxygen as the main elements. Small amounts of C were also detected at impurity levels. The carbon impurities may originate from the solution during powder formation.

**Figure 3:**
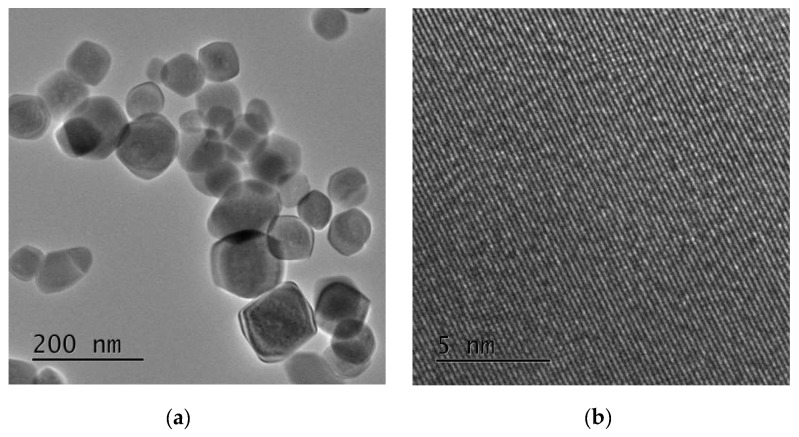
(**a**) Transmission electron microscopy (TEM) and (**b**) High resolution transmission electron microscopy HRTEM images of iron oxide nanoparticles synthesized.

TEM images of the nanoparticles are presented in Figure 3. The synthesized nanoparticles are almost hexagonal in shape. The average crystallite size is about 50 nm, which is in agreement with the value indicated by XRD analysis. The lattice fringes as seen in the high resolution transmission electron microscopy HRTEM image (Figure 3b) indicate clearly the high crystallinity of the grains.

### 3.2. Sensing Properties

The synthesized α-Fe_2_O_3_ nanoparticles were used as the sensing layer on devices with a planar-layer configuration. It can be assumed that the three-dimensional network of the iron oxide nanoparticles deposited and randomly oriented over the electrodes is responsible for the electrical paths between the adjacent Pt electrodes.

**Figure 4:**
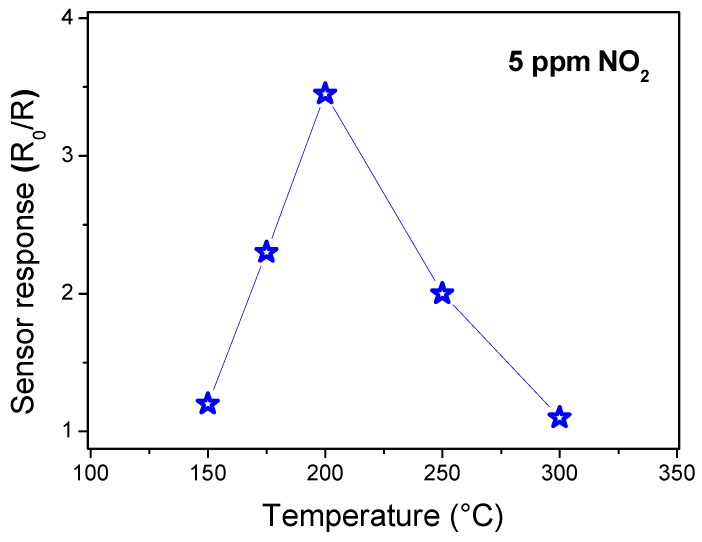
Response to 5 ppm NO_2_ of the α-Fe_2_O_3_ nanoparticles as a function of the temperature.

Before being exposed to the target gas, α-Fe_2_O_3_ sensor must reach a stable resistance, taken as R_0_. First, the optimal operating temperature of the sensors was evaluated. The operating temperature is an important parameter for a semiconductor metal oxide sensor as it determines the sensitivity of the device and can be used also to modulate the selectivity towards different gases. The temperature-dependent response measurements to 5 ppm NO_2_ gas was performed from 150 to 300 °C, as seen in Figure 4. At low temperatures, the response is restricted by the kinetics of the chemical reaction, and at high temperatures, by gas molecular diffusion. At intermediate temperatures, the two processes’ kinetics become equal, leading to the maximization of the sensor response [35]. From these results, we concluded that the operating temperature is around 200 °C. At this temperature, the sensor response presents a maximum value of 3.4 for 5 ppm of NO_2_.

It is well known that α-Fe_2_O_3_ gas sensors have been used for many applications, such as for detecting NO, NO_2_, NH_3_, SO_2_, H_2_, H_2_S, CO and a variety of volatile organic compounds (VOCs), such as ethanol, methanol, and acetone. Consequently, it is very important to do further studies on the selectivity of our developed α-Fe_2_O_3_ sensor, i.e., testing the sensors with these other gaseous substances. For this purpose, we have tested three environmental pollutant gases: NO_2_, CO, and CO_2_.

**Figure 5:**
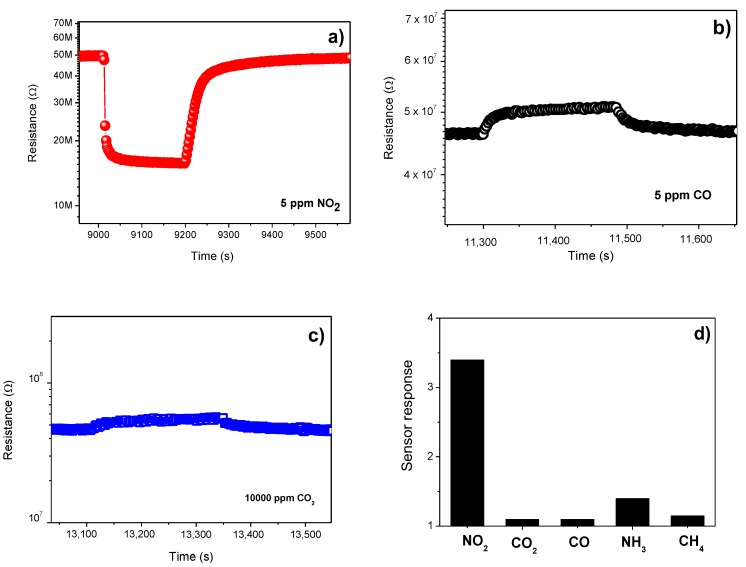
Response of α-Fe_2_O_3_ sensor to (**a**) NO_2_; (**b**) CO; (**c**) CO_2_; (**d**) Selectivity pattern to different gases at 200 °C.

Figure 5a–c displays the response of the sensor to these target gases. As can be seen, the α-Fe_2_O_3_ sensor exhibits a high sensitivity regarding NO_2_. Instead, for CO and CO_2_, the sensor sensitivity is too low. Figure 5d shows the bar chart of selectivity of the sensor. Responses to other gases, i.e., NH_3_ and methane, are also added. Tests revealed that the sensor response to 5 ppm NO_2_ was higher than that of other gases. One can conclude that the α-Fe_2_O_3_-based sensor has a good selectivity to NO_2_ gas. This finding is in agreement with the results of Navale et al. [22]. They have exposed the α-Fe_2_O_3_-based sensor to different gases, such as NO_2_, H_2_S, CH_3_OH, C_2_H_5_OH, and NH_3_, and noticed the large response to NO_2_ with respect to other gases. This remarkable selectivity of the α-Fe_2_O_3_-based sensor may be due to the high affinity of NO_2_ for hematite at the operating temperature of the sensor.

From Table 1, we can observe that the realized α-Fe_2_O_3_ sensor has the largest response towards NO_2_ compared to sensors reported by other authors. A response, (ΔR/R_0_)% of 59.9 at 1 ppm, was obtained, which indicates the high sensitivity of our device as an NO_2_ sensor.

To formulate a hypothesis on the sensing mechanism, we consider here that for semiconducting metal oxide sensors, it is usually based on the sensor resistance change with the adsorbed gas [40,41]. When the sensor surface is exposed to air, oxygen molecules are adsorbed on the surface, to be ionized by electrons trapped from the semiconductor conduction band to produce oxygen ions such as O_2_^−^, O^−^ or O^2−^. In the case of the reducing gas, the latter reacts with oxygen ions to release the captured electrons back to the semiconductor conduction band, causing the reduction in the resistance in the n-type metal oxide semiconductor or increasing the resistance in the p-type one, while in the oxidizing gas, more electrons are trapped from the conduction band. In this situation, the resistance is enhanced in an n-type semiconductor or reduced in the p-type semiconductor.

According to Figure 5, since NO_2_ is an oxidation gas and both CO and CO_2_ are reducing gases, the behavior of the resistance suggests that the synthesized α-Fe_2_O_3_ behave as a p-type semiconductor. This seems to be in contradiction with the commonly reported n-type behavior of α-Fe_2_O_3_. In fact, it is well established that, due to the oxygen deficiency, α-Fe_2_O_3_ is a native n-type semiconductor. Moreover, α-F_2_O_3_ has been known to exhibits an n- to p-type transition behavior. This intriguing phenomenon was a serious concern, particularly for gas-sensing studies [42]. This n- to p-type transition is ascribed to the formation of a surface inversion layer due to oxygen adsorption causing an increase in the holes concentration [42].

**Figure 6:**
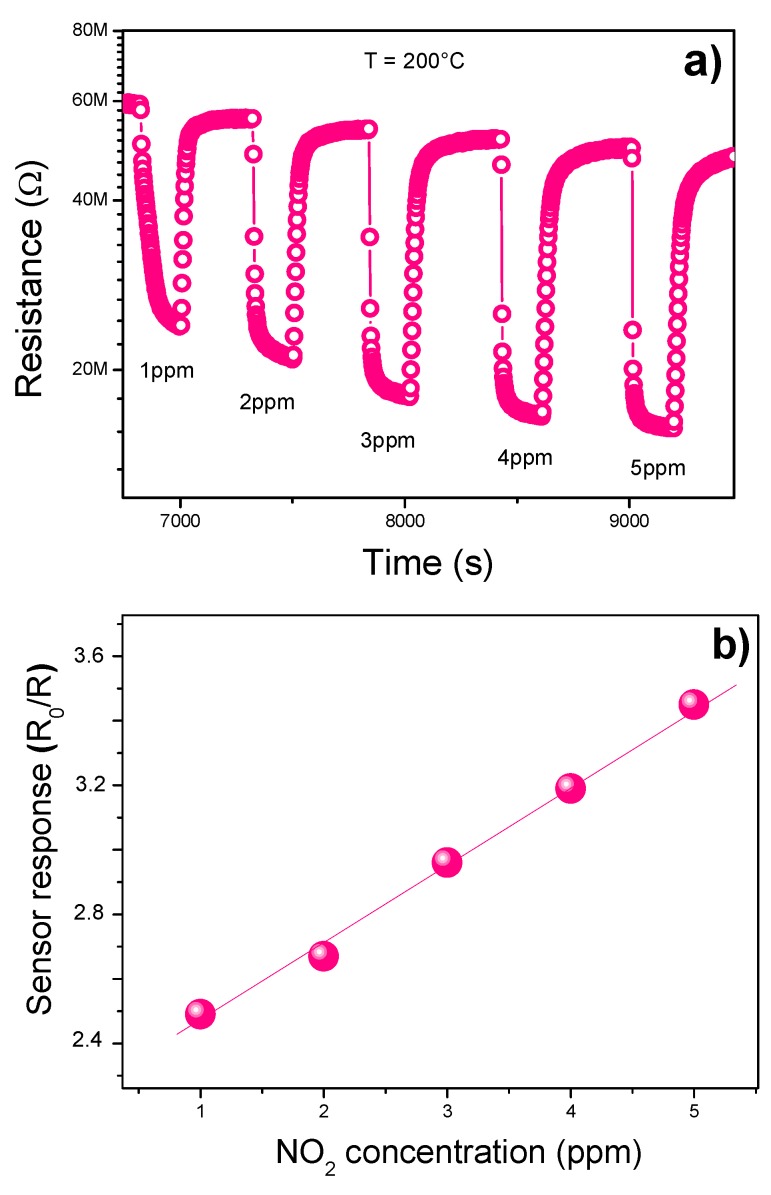
(**a**) Response of α-Fe_2_O_3_ sensor as a function of NO_2_ conc. at 200 °C. (**b**) Calibration curve.

In Figure 6a we have plotted the dynamic response of the sensor to NO_2_ exposed to different concentrations from 1 to 5 ppm.

In the investigated concentration range, the sensitivity varies almost linearly with the gas concentration, as shown in Figure 6b. As can be seen, the sensor remains highly sensitive to a low concentration of gas up to 1 ppm, suggesting that it is able to detect lower NO_2_ concentrations (in the ppb range) so as to be indicated for the sensing of nitrogen dioxide in the environment [3].

**Figure 7:**
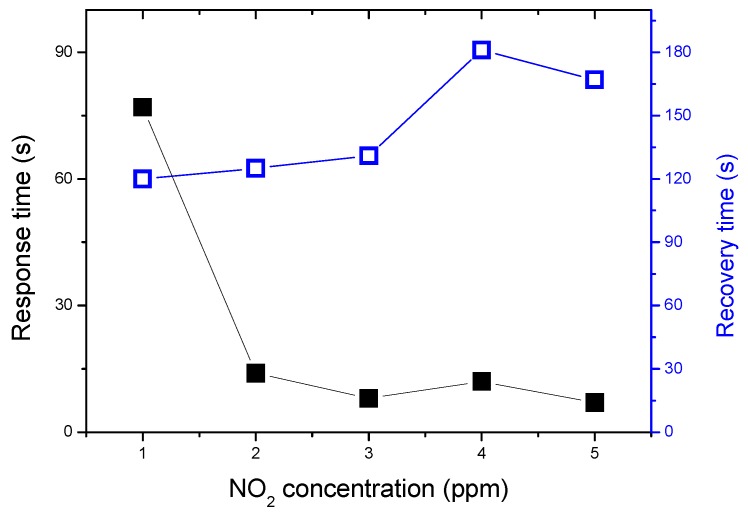
Response and recovery times of α-Fe_2_O_3_ sensor as a function of NO_2_ concentrations at the operating temperature of 200 °C.

The response and recovery times of the α-Fe_2_O_3_ sensor as a function of NO_2_ concentrations at the operating temperature of 200 °C are presented in Figure 7. The measured response times and recovery times are fast. The response time is reduced from 72 to 10 s with increasing the gas concentration from 1 to 5 ppm, this variation is due to the increase in the gas adsorption with the concentration. However, the recovery time, which depends on the gas desorption kinetic, has a reverse behavior: it increases from 8 to 180 s with the concentration. Navale et al. [22] has observed the same trend in the response and the recovery time variations as a function of NO_2_ concentration. Recently, Zhang et al. [39] have reported a longer time response of up to 2.6 min at an operating temperature of 125 °C and 4.6 min at an operating temperature at 100 °C. These large response times are mainly due to the gas adsorption kinetic slowing with a reduction in the operating temperature.

**Figure 8:**
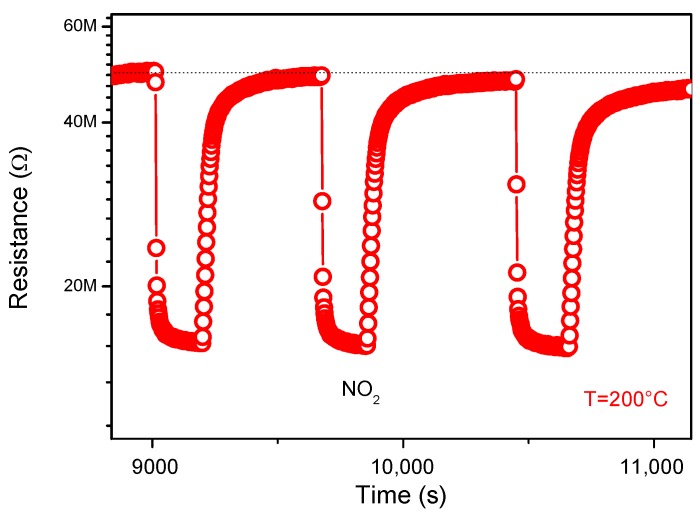
Reproducibility tests to NO_2_ gas.

Reproducibility and stability are important parameters for the performance of the sensor. Figure 8 shows, furthermore, the reproducibility of the sensor when exposed to four consecutive pulses of 5 ppm of NO_2_ gas. As clearly shown in the figure, the response of the material is almost constant. This confirms the reproducibility of the sensor material.

To check the stability of the sensor, the resistance change was studied after one month, and we observed for any changes in the behavior of the material resistance.

## 4. Conclusions

Hematite Fe_2_O_3_ nanoparticles were successfully prepared by hydrothermal technique. Crystallinity, particle size, and morphology of nanopowders were characterized by XRD, TEM, and SEM respectively. Results demonstrated that α-Fe_2_O_3_ nanoparticles synthesized by hydrothermal route are highly promising materials for NO_2_ detection at operating temperature of 200 °C. Moreover, the realized sensor is selective against a variety of interfering gases and show good characteristics of fast response/recovery times and reproducibility.

## Figures and Tables

**Table 1:** Deposition techniques, operating temperatures, and gas sensitivity of α-Fe_2_O_3_-based, NO_2_ sensors reported in literature.

Material	Target Gas	Operating Temperature (°)	Tested Gases	Preparation Technique	Response to NO_2_ (ΔR/R_0_)%	Ref.
α-Fe_2_O_3_	NO_2_	200	NO_2_, H_2_S, acetone methanol, NH_3_	Sol-gel	17.2 (200 ppm)	[22]
α-Fe_2_O_3_	Ethanol	225	NO_2_, CO, acetone, CO_2_, NH_3_, H_2_, O_2_	Sol-gel	90 (100 ppm)	[36]
Polypyrrole/α-Fe_2_O_3_	NO_2_	200	NO_2_, NH_3_, ethanol, H_2_S, methanol, Cl_2_	Sol-gel	54 (100 ppm)	[37]
graphene/α-Fe_2_O_3_	NO_2_	120	NO_2_	Hydrothermal	8.2 (5 ppm)	[38]
α-Fe_2_O_3_	NO_2_	200	NO_2_, H_2_S, acetone methanol, NH_3_	Spray pyrolysis	17.2 (200 ppm)	[39]
α-Fe_2_O_3_	NO_2_	200	NO_2_, CO, CO_2_	Hydrothermal	59.9 (1 ppm)	This work
